# Interleukin‐6 deficiency causes tissue‐specific changes in signaling pathways in response to high‐fat diet and physical activity

**DOI:** 10.14814/phy2.12064

**Published:** 2014-07-04

**Authors:** Jessica L. Sarvas, Sarah Niccoli, Eric Walser, Neelam Khaper, Simon J. Lees

**Affiliations:** 1 Medical Sciences Division, Northern Ontario School of Medicine, Thunder Bay, Ontario, Canada; 2 Department of Biology, Lakehead University, Thunder Bay, Ontario, Canada; 3 Department of Physiology and Pharmacology, University of Western Ontario, London, Ontario, Canada; 4 Health and Exercise Science, Colorado State University, Fort Collins, Colorado

**Keywords:** Insulin resistance, insulin sensitivity, type 2 diabetes

## Abstract

This study was designed to investigate the role of interleukin‐6 (IL‐6) on high‐fat diet (HFD)‐induced glucose intolerance, and the response to voluntary physical activity in the prevention of insulin resistance. Six‐week‐old wild‐type (WT) and IL‐6 knockout (KO) mice with (RUN) or without (SED) access to running wheels were fed a HFD (60% from kcal) for 4 weeks. A glucose tolerance test revealed that blood glucose levels were 25–30% higher in KO RUN compared to all other groups. In WT RUN, weight gain was positively correlated with total caloric intake; however, this correlation was absent in KO RUN. In soleus muscle, there was a 2‐fold increase in SOCS3 expression in KO RUN compared to all other groups. In gastrocnemius and plantaris muscles, Akt phosphorylation was 31% higher in WT RUN compared to WT SED, but this effect of running was absent in KO mice. Additionally, there was a 2.4‐fold increase in leptin expression in KO RUN compared to KO SED in the gastrocnemius and plantaris muscles. In the liver, there was a 2‐ to 3.8‐fold increase in SOCS3 expression in KO SED compared to all other groups, and AMPK*α* phosphorylation was 27% higher in WT mice (both RUN and SED) compared to KO mice (both RUN and SED). This study provides new insights into the role of the IL‐6 in metabolism and energy storage, and highlights tissue‐specific changes in early signaling pathways in response to HFD for 4 weeks. The collective findings suggest that endogenous IL‐6 is important for the prevention of insulin resistance leading to type 2 diabetes.

## Abstract (precis)

This study was designed to investigate the role of interleukin‐6 (IL‐6) on high‐fat diet (HFD)‐induced glucose intolerance, and the response to voluntary physical activity in the prevention of insulin resistance. This study provides new insight into the role of the IL‐6 in metabolism and energy storage, and highlights tissue‐specific changes in early signaling pathways in response to HFD for 4 weeks. The collective findings suggest that endogenous IL‐6 is important for the prevention of insulin resistance leading to type 2 diabetes.

## Introduction

Physical inactivity has become the fourth leading risk for global death in recent years, and has led to increased obesity (World Health Organization). Obesity has been linked to 3.4 million deaths each year, and along with increased sedentary lifestyle, can be caused by improper nutrition, including increased consumption of high‐fat foods (Calder et al. 2011). In obesity, increased numbers of macrophages infiltrate the white adipose tissue, which causes increased production of inflammatory cytokines (Weisberg et al. 2003). Obesity has been characterized as a state of chronic low‐grade inflammation, due to the increased secretion and subsequent ~2‐ to 3‐fold elevation in systemic inflammatory markers, including tumor necrosis factor‐*α*, interleukin‐1*β* (IL‐1 *β*), and interleukin‐6 (IL‐6) (Kern et al. 2001; Weisberg et al. 2003). Several pathological conditions, including type 2 diabetes, are associated with obesity leading to increased morbidity and mortality (World Health Organization). The altered function of insulin at peripheral tissues leads to insulin resistance in skeletal muscle, liver, and adipose tissue, and this is critical to the development of impaired glucose metabolism and the progression of type 2 diabetes (Rev. in Sarvas et al. 2013).

IL‐6 is a pleiotropic cytokine that is secreted by and acts on a wide variety of tissues, and exhibits both pro‐ and anti‐inflammatory properties (Starkie et al. 2003; Steensberg et al. 2003). The plasma level of IL‐6 in healthy humans is typically less than 5 pg mL^−1^ (Kado et al. 1999). Several studies, both *in vitro* and *in vivo*, have shown that IL‐6 and various other cytokines increase the phosphorylation of signal transducer and activator of transcription (STAT) proteins (Heinrich et al. 1998). Activated STAT3 is translocated to the nucleus, where it is able to regulate the transcription of IL‐6 target genes (Carey et al. 2006; Glund et al. 2007; Begue et al. 2013; Kim et al. 2013). IL‐6‐mediated Jak/STAT signaling can be induced rapidly, and results in increased phosphorylation of STAT3 under inflammatory conditions. Activated STAT3 proteins induce the expression of suppressor of cytokine signaling (SOCS) proteins, which in turn acts as a negative feedback on STAT3 activation, and as a negative regulator of insulin signaling and glucose metabolism in tissues (reviewed in Sarvas et al. 2013). The ability of SOCS3 to inhibit insulin signaling suggests that these proteins influence energy balance and glucose homeostasis within the body. In support of this, SOCS3 is also known to have a role in the development of leptin resistance. Leptin is a hormone secreted by adipocytes that regulates energy balance and caloric intake in the body. In the hypothalamus, leptin signaling results in increased expression of neuropeptides, which cause increased energy expenditure and appetite suppression. In the peripheral tissues, leptin expression results in increased activity of AMP‐activated protein kinase (AMPK) under normal conditions (Wolsk et al. 2011). AMPK phosphorylates target proteins leading to increased fatty acid oxidation, glucose transport, and lipolysis in skeletal muscle, liver, and adipose tissue (Martin et al. 2006).

Chronically elevated IL‐6 mediates inhibitory effects on insulin signaling and glucose metabolism, and therefore, has been linked to insulin resistance in peripheral tissues (reviewed in Sarvas et al. 2013). Interestingly, IL‐6‐deficient mice also become obese and insulin resistant after 6 months of age (Wallenius et al. 2002a,b). In contrast to chronically altered IL‐6, acute increases in this cytokine are observed in response to other stimuli, including exercise. An acute and transient increase in circulating inflammatory markers occur during, and remain elevated up to 4 h after exercise. It has been reported that plasma IL‐6 concentrations can increase up to 100‐fold (Ostrowski et al. 1999). Increases in IL‐6 mRNA and protein have been reported in skeletal muscle during exercise, and it has been concluded that skeletal muscle cells produce enough IL‐6 to account for the large increase in plasma IL‐6 levels (Allen et al. 2010; Pedersen and Febbraio 2012). It has been well documented that regular exercise can alleviate or protect against type 2 diabetes by enhancing insulin sensitivity in peripheral tissues, and it can protect against insulin resistance in the high‐fat (35.5–60% from kcal) feeding model (Bradley et al. 2008). In fact, regular exercise is more effective than current pharmacological treatments for type 2 diabetes (Bradley et al. 2008; Hawley and Lessard 2008; Sharoff et al. 2010). Therefore, an IL‐6 paradox does exist, such that elevated IL‐6 can lead to the development of insulin resistance, and yet may also lead to increased insulin sensitivity.

Understanding the differences between chronic and acute increases in circulating IL‐6 may be important to its effects on target tissues, and particularly, the effects on insulin signaling within these tissues (reviewed in Sarvas et al. 2013). An improved understanding of the mechanisms behind IL‐6 signaling within the complex model of high‐fat diet (HFD) and physical activity is required to develop improved treatment strategies for type 2 diabetes. The purpose of this study was to determine both the role of IL‐6 on HFD induced glucose intolerance, and in response to voluntary physical activity in the prevention of insulin resistance in IL‐6‐deficient mice.

## Research Design and Methods

### Animals

**Figure 1.**
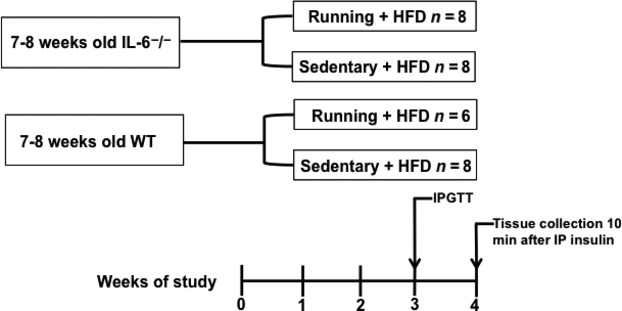
Flowchart of the study design. HFD, high‐fat diet; IPGTT, intraperitoneal glucose tolerance test; IP, intraperitoneal; WT, wild type.

The IL‐6 knockout (KO) mice (B6.129S2‐Il6^t*m1Kopf*^/J) were purchased from Jackson Laboratory (Bar Harbour, ME). Male KO (*n* = 16), and wild type (WT) (*n* = 14) were obtained at ~6 weeks of age, and studied after 1 week of acclimatization (Fig. 1). Mice were housed under controlled temperature (18–20°C), humidity (40–70%), decibel level (<70 dB), and lighting (12 h of light; 12 h of dark) with free access to food and water. All animal experiments were performed in accordance with the institutional animal care committee guidelines at Lakehead University.

### Experimental protocol

The mice were fed a HFD (D12492; Research Diets Inc., New Brunswick, NJ) containing the following nutrient content (in kcal percent): fat 60%, protein 20%, carbohydrate 20%; with a total caloric content of 5.24 kcal·g^−1^. Eight KO and six WT mice had free access to running wheels. Over the 4 weeks of the study, food intake and running distance were recorded daily, and body weight was recorded weekly. Daily running distances were tracked using CatEye Velo 5 (CatEye America, Boulder, CO). 

### Glucose tolerance test

After 3 weeks of HFD, mice were morning fasted for 5 h prior to the glucose tolerance test (GTT) (Fig. 1). Baseline blood glucose levels were taken prior to a bolus intraperitoneal injection of glucose (1 mg·kg^−1^). Blood samples were taken from the tail vein at baseline (0), and after 30, 60, 90, and 120 min. Glucose levels were measured with a handheld whole‐blood glucose monitor (OneTouch Ultra2, LifeScan Canada, Burnaby, BC, Canada). The blood glucose response was quantified by calculating the area under the curve after the injection of glucose and subtracting baseline to describe the concentration of glucose in the blood 120 min after administration.

### Tissue collection

At the end of the 4 weeks, mice were morning fasted for 5 h, and then insulin was administered intraperitoneally to all animals at a dose of 10 U·kg^−1^. It was confirmed that the insulin treatment was sufficient for stimulating insulin signaling at peripheral tissues by running a western blot and probing for Akt phosphorylation. There was an approximately 8.5‐fold difference in Akt phosphorylation between muscles stimulated with a high (10 U·kg^−1^) and low (0.5 U·kg^−1^) insulin dose (data not shown). Ten minutes after the insulin injection, mice were anesthetized with isofluorane, and the hearts were removed (Fig. 1). The liver, soleus, and gastrocnemius/plantaris (combined) muscles (left and right leg muscles) were removed and immediately frozen in liquid nitrogen for further analysis. The gastrocnemius and plantaris muscles were combined because they both have similar type I and type II myosin heavy chain profiles (both have a predominant IIB, with varying type IIA and IIX), whereas the soleus is approximately 50% type I myosin heavy chain and is postural.

### Tissue lysis

Frozen muscle and liver tissues were homogenized in ice‐cold lysis buffer (25 mmol·L^−1^ Tris pH = 7.5, 150 mmol·L^−1^ NaCl, 1 mmol·L^−1^ EDTA, 1% Triton‐X 100) supplemented with protease and phosphatase inhibitors. The tissues were disrupted using the Qiagen TissueLyser (Qiagen, Toronto, ON, Canada). Samples were centrifuged at 16,000 × *g* for 10 min at 4°C, and then the supernatants were collected and stored at −80°C for immunoblot and ELISA analysis.

### Western blot analysis

A total of 45 *μ*g of protein was loaded and resolved by SDS‐PAGE on 15% polyacrylamide gels and transferred to nitrocellulose membranes. Immunoblotting was performed using the following primary antibodies: phosphorylated Akt^SER473^, leptin (Abcam, Cambridge, MA); pan Akt, SOCS3, phosphorylated AMPK*α*^THR172^, and total AMPK*α* (Cell Signaling Technology, Danvers, MA). After incubation with goat‐anti rabbit (HRP) secondary antibody (Thermo Scientific Pierce, Rockford, IL), the immunoreactive complexes were detected with enhanced chemiluminescence (ChemiDoc^™^ XRS, Bio‐Rad, Hercules, CA) and quantified by densitometry using ImageJ software. Each western was normalized to a loading control sample. This positive control varied depending on the target protein of the blot. The specific controls were as follows: pAkt/total Akt (C2C12 myotubes treated with 100 nmol·L^−1^ dose of humulin R to mimic an acute dose of insulin for 15 min prior to cell lysis), pAMPK*α*/total AMPK*α* (C2C12 myotubes treated with 1 mmol·L^−1^ AICAR for 24 h prior to cell lysis), and SOCS3/leptin (C2C12 myotubes treated with 100 nmol·L^−1^ dose of humulin R for 3 days to mimic a chronic dose of insulin prior to cell lysis).

### Enzyme‐linked immunosorbent assay

To quantify the endogenous levels of phosphorylated STAT3 in soleus, gastrocnemius, plantaris, and liver tissue, PathScan^®^ Phospho‐Stat3 (Tyr705) Sandwich ELISA Kit (Cell Signaling Technology) was used as per manufacturer's manual.

### Statistics

Prior to determining animal numbers, a power analysis was performed. Data are presented as means ± SEM. Comparisons between groups were done using two‐way analysis of variance (ANOVA) for all comparisons, except average daily running distance (one‐way ANOVA), and the correlation of change in body weight and caloric intake. ANOVA tests were followed by Fisher LSD post hoc tests (SigmaStat software, Systat, Chicago, IL). Significance was accepted at *P *≤**0.05.

## Results

### Sedentary mice showed increased weight gain compared to runners

**Figure 2.**
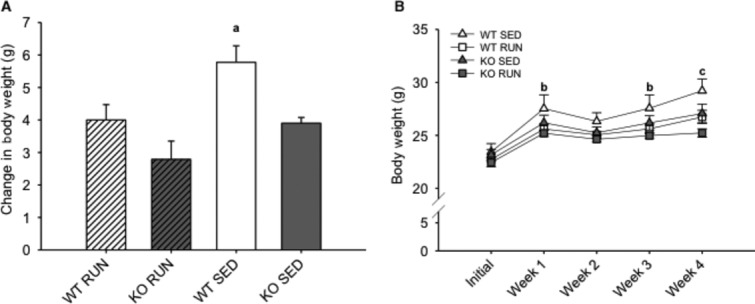
Sedentary mice showed increased weight gain compared to runners after 4 weeks on a high‐fat diet. (A) The mean change in body weight (grams) in wild‐type (WT) and IL‐6 knockout (KO) groups with (RUN) and without (SED) voluntary access to running wheels fed a high‐fat diet for 4 weeks. (B) The mean body weight (grams) in WT and KO groups (RUN & SED) over 4 weeks on a high‐fat diet. ^a^Denotes significant differences (*P* ≤ 0.05) between WT SED and all other groups. ^b^Denotes significant differences (*P* ≤ 0.05) between WT SED and KO RUN groups. ^c^Denotes significant differences (*P* ≤ 0.05) between WT SED and all other groups. Data are presented as mean ± SEM, (*n* = 6–8 per group).

Initial body weights of WT and KO mice, in both running (RUN) and sedentary (SED) groups were taken, and then recorded weekly for 4 weeks following the introduction of HFD (Fig. 2A). There were no differences in body weight between groups at the beginning of the study. As expected, WT SED mice gained more weight compared to RUN mice (24.8 vs. 17.6%, *P* < 0.05) (Fig. 2A). However, the KO mice showed no differences in weight gain between the SED and RUN groups (*P* = 0.08) (Fig. 2A). Within WT, WT SED mice gained more weight compared to KO SED mice (24.8 vs. 16.8%, *P* < 0.05), but there were no differences in weight gain between the WT and KO mice in the RUN group (*P* = 0.08) (Fig. 2A). Differences in body weight were observed between WT SED and KO RUN mice at weeks 1, 3, and 4 (*P* < 0.05) (Fig. 2B). At week 4, WT SED mice had higher body weight compared to all other groups (*P* < 0.05) (Fig. 2B).

### Runners consumed more calories compared to sedentary mice

**Figure 3.**
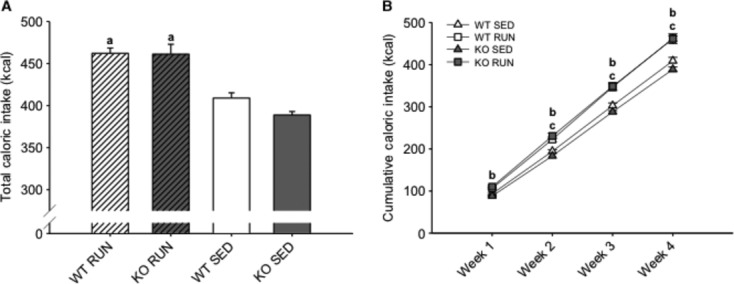
Runners consumed more calories compared to sedentary mice over 4 weeks on a high‐fat diet. (A) The mean total caloric intake (kcal) for wild‐type (WT) and IL‐6 knockout (KO) groups with (RUN) and without (SED) voluntary access to running wheels fed a high‐fat diet for 4 weeks. (B) The mean cumulative caloric intake (kcal) for WT and KO groups (RUN & SED) over 4 weeks on a high‐fat diet. ^a^Denotes significant differences (*P* ≤ 0.05) between RUN and SED mice. ^b^Denotes significant differences (*P* ≤ 0.05) between KO RUN and both KO SED and WT SED groups. ^c^Denotes significant differences (*P* ≤ 0.05) between WT RUN and both KO SED and WT SED groups. Data are presented as mean ± SEM, (*n* = 6–8 per group).

**Figure 4.**
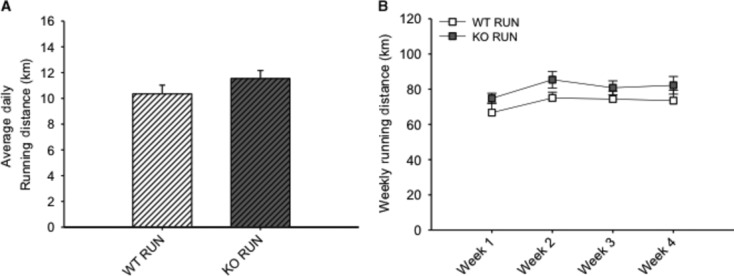
There were no differences in running distances between groups. (A) The average daily running distance (km) for wild‐type (WT) and IL‐6 knockout (KO) groups with (RUN) voluntary access to running wheels fed a high‐fat diet for 4 weeks. (B) The mean weekly running distance (km) each week for WT and KO RUN groups fed a high‐fat diet for 4 weeks. Data are presented as mean ± SEM, (*n* = 6–8 per group).

Daily food intake was recorded over 4 weeks, and caloric intake of HFD was calculated (Fig. 3A and B). It was found that RUN mice consumed more calories compared to SED mice in both WT (13.0%, *P* < 0.05) and KO (18.6%, *P* < 0.05) groups (Fig 3A). By week 2, both KO RUN and WT RUN mice had increased cumulative caloric intake compared to both KO SED and WT SED mice (*P* < 0.05), and this increase was also observed at weeks 3 and 4 (Fig. 3B). There were no differences in average daily running distance between these two groups over the duration of the study (10.3 km day^−1^ ± 0.7 vs. 11.5 km day^−1^ ± 0.6, for WT and KO mice, respectively) (*P* = 0.083) (Fig. 4A), and running distances remained constant over the 4 weeks (Fig. 4B).

### Lack of IL‐6 negatively affects glucose tolerance in runners

**Figure 5.**
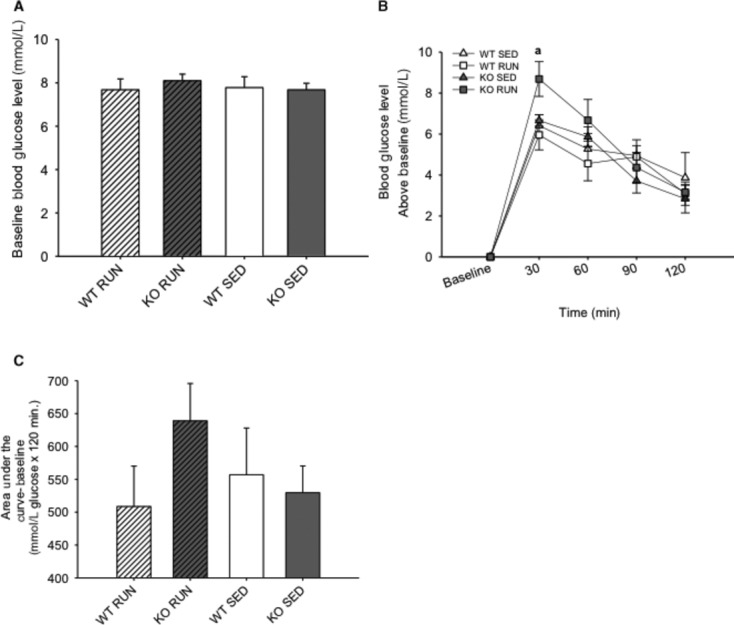
Lack of IL‐6 negatively affects glucose tolerance in runners. (A) The mean fasting (baseline) blood glucose level (mmol·L^−1^) for wild‐type (WT) and IL‐6 knockout (KO) groups with (RUN) and without (SED) voluntary access to running wheels after 3 weeks on a high‐fat diet. (B) The mean blood glucose levels above baseline (mmol·L^−1^) following a bolus intraperitoneal injection of glucose in WT and KO groups (RUN & SED) after 3 weeks on a high‐fat diet. (C) The mean glucose area under the concentration–time curve above baseline (mmol·L^−1^ glucose × 120 min) for WT and KO groups (RUN & SED) after 3 weeks on a high‐fat diet. ^a^Denotes significant differences (*P* ≤ 0.05) between KO RUN and all other groups. Data are presented as mean ± SEM, (*n* = 6–8 per group).

After exposure to HFD for 3 weeks, there were no differences in fasting blood glucose levels between the groups (Fig. 5A). In order to test glucose tolerance, mice were given a bolus intraperitoneal injection of glucose and circulating glucose concentration was measured every 30 min for 2 h. After 30 min, blood glucose levels were 25–30% higher (*P* < 0.05) in the KO RUN compared to all other groups (Fig. 5B). The calculated area under the curve for the GTT yielded a nonsignificant (25.6%) increase in blood glucose in KO RUN compared to WT RUN (639 ± 56.6 vs. 508.7 ± 66.9, *P* = 0.15) (Fig. 5C).

### Lack of IL‐6 disrupts the link between caloric intake and weight gain in runners

**Figure 6.**
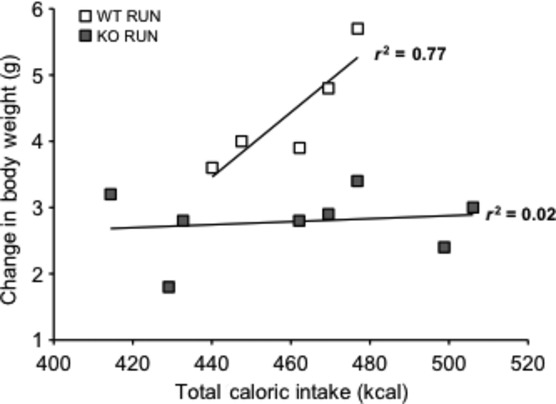
Lack of IL‐6 disrupts the relationship between caloric intake and weight gain in runners. The correlation between total caloric intake (kcal) and total change in body weight (grams) in wild‐type (WT) (*n* = 5) and IL‐6 knockout (KO) (*n* = 8) groups with (RUN) voluntary access to running wheels fed a high‐fat diet for 4 weeks. The data from one WT animal were removed as an outlier based on the value being greater than two standard deviations from the mean.

The correlation between total caloric intake and weight gain was examined between WT RUN and KO RUN mice. The relationship between caloric intake and weight gain is not reported in sedentary animals of either genotype because they were group housed, and therefore, no individual food intakes were recorded. As expected, the amount of weight gain was positively correlated (*r*^2^ = 0.77) with total caloric intake in WT RUN mice over 4 weeks on HFD (Fig. 6). However, this association was absent in KO RUN mice (*r*^2^ = 0.02), indicating an uncoupling of the caloric intake and weight gain relationship.

### Phosphorylation and expression of signaling proteins

**Figure 7.**
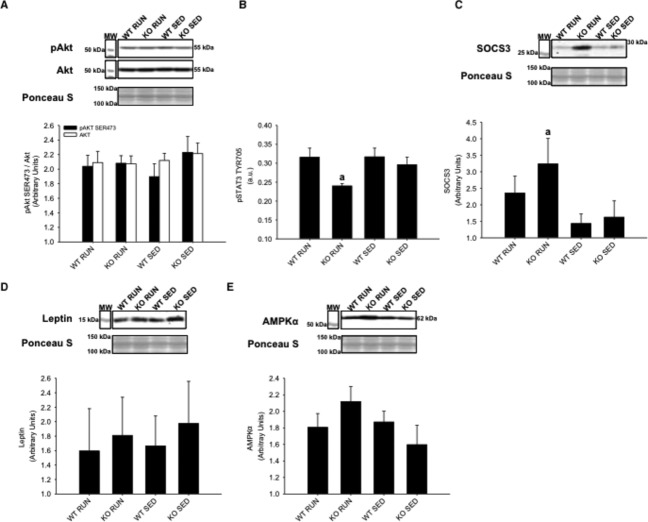
Soleus skeletal muscle protein expression of Akt, STAT3, SOCS3, leptin, and AMPK*α* from wild‐type (WT) and IL‐6 knockout (KO) groups with (RUN) and without (SED) voluntary access to running wheels fed a high‐fat diet for 4 weeks. Insulin‐stimulated (A) Akt^SER^^473^ phosphorylation (B) STAT3^TYR^^706^ phosphorylation (C) SOCS3 expression (D) Leptin expression (E) AMPK*α* expression. ^a^Denotes significant differences (*P* ≤ 0.05) between KO RUN and all other groups. Data are presented as mean ± SEM, (*n* = 6–8 per group). Ponceau S stains are shown as markers of equal protein loading.

After 4 weeks on HFD, the phosphorylation and abundance of signaling proteins associated with insulin resistance, physical activity, and IL‐6 were determined in soleus muscle, gastrocnemius/plantaris muscles, and liver tissue. A bolus intraperitoneal injection of insulin was administered 10 min prior to tissue collection. In the soleus muscle, no differences in insulin stimulated Akt^SER473^ phosphorylation were found between groups (Fig. 7A). In soleus muscle, STAT3^TYR706^ phosphorylation was decreased in KO RUN group compared to all other groups (*P* < 0.05) (Fig. 7B). Increased SOCS3 was found in the KO RUN group compared to all other groups (*P* < 0.05), supporting its inhibitory role for STAT3 (Fig. 7C). It was found that leptin expression did not differ between groups in soleus muscle (Fig. 7D). AMPK*α*^THR172^ phosphorylation was not detected in any of the groups, and there were no differences in AMPK*α* expression between the groups (Fig. 7E).

**Figure 8.**
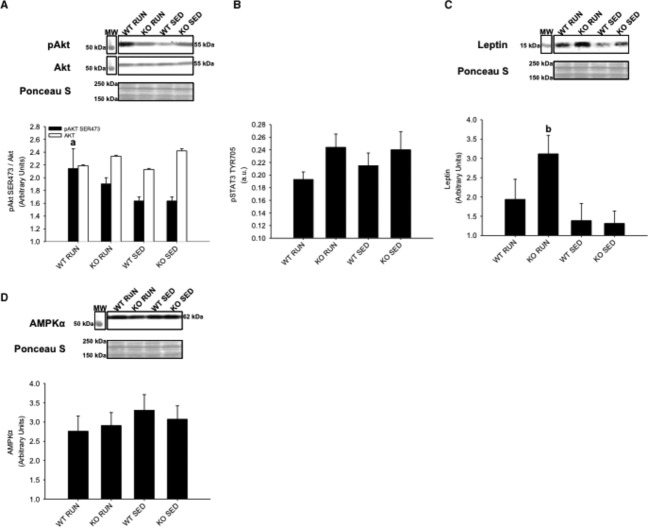
Gastrocnemius and plantaris skeletal muscle protein expression of Akt, STAT3, leptin, and AMPK*α* from wild‐type (WT) and IL‐6 knockout (KO) groups with (RUN) and without (SED) voluntary access to running wheels fed a high‐fat diet for 4 weeks. Insulin‐stimulated (A) Akt^SER^^473^ phosphorylation (B) STAT3^TYR^^706^ phosphorylation (C) Leptin expression (D) AMPK*α* expression. ^a^Denotes significant differences (*P* ≤ 0.05) between WT RUN and WT SED groups. ^b^Denotes significant differences (*P* ≤ 0.05) between KO RUN and KO SED groups. Data are presented as mean ± SEM, (*n* = 6–8 per group). Ponceau S stains are shown as markers of equal protein loading.

In contrast to soleus muscle, insulin‐stimulated Akt^SER473^ phosphorylation was increased in WT RUN compared to WT SED in gastrocnemius/plantaris muscles (*P* < 0.05) (Fig. 8A). There were no differences in STAT3^TYR706^ phosphorylation between groups (Fig. 8B), and SOCS3 expression was not detected in any of the groups. However, leptin expression was increased in KO RUN compared to KO SED in gastrocnemius/plantaris muscles (*P* < 0.05) (Fig. 8C). Similar to soleus muscle, AMPK*α*^THR172^ phosphorylation was not detected in any of the groups, and there were no differences in AMPK*α* expression between groups (Fig. 8D).

**Figure 9.**
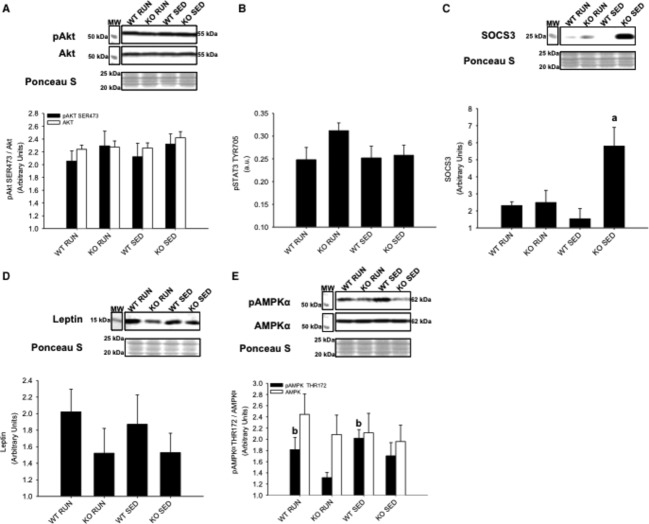
Liver protein expression of Akt, STAT3, SOCS3, leptin, and AMPK*α* from wild‐type (WT) and IL‐6 knockout (KO) groups with (RUN) and without (SED) voluntary access to running wheels fed a high‐fat diet for 4 weeks. Insulin‐stimulated (A) Akt^SER^^473^ phosphorylation (B) STAT3^TYR^^706^ phosphorylation (C) SOCS3 expression (D) Leptin expression (E) AMPK*α*^THR^^172^ phosphorylation. ^a^Denotes significant differences (*P* ≤ 0.05) between KO SED and all other groups. ^b^Denotes significant differences (*P* ≤ 0.05) between WT and KO groups. Data are presented as mean ± SEM, (*n* = 6–8 per group). Ponceau S stains are shown as markers of equal protein loading.

In liver, there were no differences in insulin‐stimulated Akt^SER473^ phosphorylation between groups (Fig. 9A). Similar to gastrocnemius/plantaris muscles, there were no differences in STAT3^TYR706^ phosphorylation between groups (Fig. 9B). However, SOCS3 expression was increased in KO SED compared to all other groups (*P* < 0.05) (Fig. 9C). No differences in leptin expression were found between groups (*P* = 0.27 for WT RUN compared to KO RUN) (Fig. 9D). Unlike the skeletal muscles, AMPK*α*^THR172^ phosphorylation was detected in liver tissue. It was found that AMPK*α*^THR172^ phosphorylation was increased in WT groups (both RUN and SED) compared to KO groups (both RUN and SED) (*P* < 0.05) (Fig. 9E).

## Discussion

It has been previously shown that IL‐6 influences glucose and lipid metabolism, and more specifically, insulin action (Wallenius et al. 2002a,b; van Hall et al. 2003). One intriguing aspect of IL‐6 is the apparent context‐dependent dual role of this cytokine in glucose metabolism (Sarvas et al. 2013). This study was designed to investigate the complex role of IL‐6 on HFD‐induced glucose intolerance, and the response to voluntary physical activity in the prevention of insulin resistance. Previous research has established that changes in the expression of SOCS3, leptin, and the activation of STAT3, and AMPK are linked to the regulation of insulin signaling in obesity and diabetes (Sarvas et al. 2013). This study was first to report that the lack of IL‐6 disrupts the link between caloric intake and weight gain in runners, which provides new insight into the role of IL‐6 in metabolism and energy storage. This study also revealed important tissue‐specific differences in SOCS3 expression, leptin expression, and Akt phosphorylation. In its entirety, the findings of this study suggest that endogenous IL‐6 is important for the prevention of insulin resistance induced by HFD.

During and up to 4 h after exercise, plasma IL‐6 concentrations can increase approximately 100‐fold, which can increase insulin sensitivity in peripheral tissues (Bradley et al. 2008; Pedersen and Febbraio 2012). However, this increase was dramatically reduced after 4 h, therefore, differences in plasma IL‐6 were not expected between WT RUN and WT SED groups since tissues were collected 5 h after running wheels were removed. Recent findings published by Benrick et al. (2012) found that IL‐6 KO mice did not benefit from running to the same extent as WT mice. Therefore, they concluded that endogenous IL‐6 contributes to exercise‐induced insulin sensitivity. While these findings are in agreement with this study, there are some differences between the studies, including observations of weight gain (Benrick et al. 2012). The study by Benrick et al. (2012) utilized a voluntary physical activity model with HFD, and they found no differences in weight gain after 5 weeks on HFD between the WT and IL‐6 KO mice, in either the sedentary or running groups. This was in contrast to this study, which found that WT SED mice had increased weight gain compared to all other groups after 4 weeks on HFD. The HFD chosen for this study contained a nutrient content of 60% fat, whereas the HFD used by Benrick et al. (2012) contained 35.5% fat, which may account for the discrepancies in weight gain findings.

Although it is known that HFD leads to increased IL‐6, both the amount and duration of this increase is variable, and can lead to differences in insulin signaling. In long‐term studies without the use of HFD, blood glucose levels were increased in IL‐6 KO mice compared to WT mice, and the IL‐6 KO mice were obese. However, abnormal glucose homeostasis and lipid metabolism do not occur until 6 months of age (Wallenius et al. 2002a,b). Previous studies that investigated the effects of HFD for 12–14 weeks also found that blood glucose levels were increased in IL‐6 KO mice compared to WT mice (Di Gregorio et al. 2004; Matthews et al. 2010). Although these long duration studies demonstrated obesity and insulin resistance in the IL‐6 KO mice, the experimental designs utilized overnight fasts and larger glucose doses to induce more robust effects. In an effort to detect the effects of IL‐6 on early changes in glucose tolerance, and to assess insulin action within a more physiological context, the GTT was conducted after 3 weeks on HFD in the present study (McGuinness et al. 2009; Ayala et al. 2010). The mice were morning fasted for 5 h to mimic an overnight fast in humans due to metabolic differences, and administered a conservative dose (1 mg·kg^−1^) of glucose prior to the test (Ayala et al. 2010). The GTT revealed that blood glucose increased 25–30% more in KO RUN compared to all other groups after 30 min. Despite running equal distances, and consuming the same amount of calories, the KO RUN exhibited early signs of glucose intolerance compared to WT RUN. This impaired glucose tolerance in KO RUN is an indicator of developing insulin resistance, and these findings suggest that IL‐6 has an important role in the beneficial effects of physical activity on HFD‐induced glucose intolerance.

IL‐6 has been shown to inhibit glycogen synthase activity and increase glycogen phosphorylase activity in rodent hepatocytes (Kanemaki et al. 1998). Additionally, IL‐6 produced by contracting skeletal muscle may mediate hepatic glucose output during exercise, and when glucose was ingested prior to exercise, IL‐6 release from skeletal muscle was attenuated (Steensberg et al. 2001; Febbraio et al. 2003). In an IL‐6 secreting tumor model, there was increased glycogen breakdown, which showed that IL‐6 has a direct effect on glycogen metabolism in the liver (Metzger et al. 1997). In the present study, the amount of weight gain was positively correlated with total caloric intake in WT RUN mice, but this correlation was absent in KO RUN mice. The lack of endogenous IL‐6 in KO RUN mice may have prevented effective glycogen breakdown in skeletal muscle and liver, which may have led to less glucose utilization during physical activity. Consequently, the KO RUN mice may have utilized available dietary fats as a main energy source. Although KO RUN were consuming similar amounts of food compared to WT RUN, utilizing fat as the primary energy substrate during physical activity may contribute to the disrupted relationship between weight gain and total caloric intake in KO RUN mice.

Another possible explanation for the disrupted relationship between weight gain and total caloric intake in KO RUN mice involves increased energy expenditure and thermogenesis in the KO RUN mice. Uncoupling proteins (UCPs) generate heat by uncoupling oxidative phosphorylation (Nedergaard et al. 2001). The increased expression of these proteins in brown adipose tissue (BAT) and white adipose tissue (WAT) result in increased energy expenditure, and are involved in temperature and body weight regulation (Nedergaard et al. 2001). It has been shown that acute increases in IL‐6 during physical activity can increase UCP1 expression in WAT, and UCP1 expression was lower in IL‐6 KO compared to WT mice (Knudsen et al. 2014). However, this physical activity‐induced increase in UCP1 expression was not completely blunted in IL‐6 KO mice. In this study, the HFD may have prevented physical activity‐induced thermogenesis in WT RUN mice due to pro‐inflammatory cytokines and other secreted factors associated with chronic low‐grade inflammation, which can inhibit the expression of UCP1 in BAT and WAT (Sakamoto et al. 2013). Therefore, increased thermogenesis may have been blunted by HFD to a greater extent in WT RUN mice compared to the KO RUN mice leading to the uncoupling of the caloric intake–weight gain relationship.

It has been established that both the biochemical adaptations to physical activity, and the effects of various dietary conditions differ between skeletal muscle types (Holloszy 1976; Blachnio‐Zabielska et al. 2010; Sharma et al. 2013). The results in this study provide novel support for different changes in early signaling pathways between oxidative and glycolytic skeletal muscles when exposed to HFD and physical activity. Increased SOCS3 expression has been associated with insulin resistance in peripheral tissues (Shi et al. 2004, 2006; Ueki et al. 2004), but it has also been shown that physical activity can lead to increased SOCS3 mRNA expression (Spangenburg et al. 2006). Spangenburg et al. (2006) found that exercise training increased SOCS3 mRNA expression in rat soleus and plantaris skeletal muscles, which may be linked to subsequent increases in IL‐6 expression. Rat plantaris and mouse soleus muscle have comparable oxidative capacities measured by succinate dehydrogenase activity (Bloemberg and Quadrilatero 2012). This study demonstrated that the lack of IL‐6 led to increased SOCS3 expression in response to physical activity in soleus muscle of KO RUN mice. If increased SOCS3 expression contributes to increased IL‐6 expression during physical activity, then KO RUN mice may have prevented a feedback mechanism on SOCS3, allowing SOCS3 expression to continue to increase in KO RUN compared to the other groups. Therefore, this study demonstrates for the first time that physical activity can induce increases in SOCS3 expression at the protein level.

Insulin stimulates the activation of Akt causing the translocation of glucose transporter 4 (GLUT‐4) vesicles to the plasma membrane, leading to glucose uptake in skeletal muscle (Sarvas et al. 2013). Physical activity increases insulin sensitivity, while HFD has been shown to cause insulin resistance. This study demonstrated that physical activity prevented insulin resistance in gastrocnemius and plantaris muscles of WT RUN mice. However, insulin‐stimulated Akt phosphorylation in KO RUN mice was not increased compared to either SED group. This finding implies that the prevention of insulin resistance in response to physical activity requires endogenous IL‐6 in skeletal muscle.

It was hypothesized that KO mice may have impaired glycogen breakdown due to the lack of IL‐6, resulting in a higher demand for free fatty acids. Since leptin can increase fatty acid uptake in skeletal muscle, the increased leptin expression in the gastrocnemius and plantaris muscles of KO RUN mice can possibly be explained due to the higher demand for free fatty acids in these muscles during physical activity (Steinberg et al. 2002; Todd et al. 2005). If KO mice have restricted glucose availability, and are mainly utilizing fats, this resembles the high‐fat and low carbohydrate formulation of ketogenic diets. Increased leptin levels have been reported in rats that were fed a ketogenic diet compared to a standard diet, and these findings provide additional support for increased leptin expression in the gastrocnemius and plantaris muscles due to the higher demand for free fatty acids (Thio et al. 2006).

Unlike in the soleus muscle, SOCS3 expression was not detected in the gastrocnemius and plantaris muscles in any of the groups. Additionally, no differences were observed in insulin‐stimulated Akt phosphorylation or leptin expression in the soleus muscle. The fiber differences among soleus, and gastrocnemius/plantaris muscles may account for the differences in SOCS3 expression, Akt phosphorylation, and leptin expression between the muscles. Mouse gastrocnemius and plantaris muscles have a much higher percentage of myosin heavy chain IIB fibers, and glycolytic capacity compared to mouse soleus muscle (Bloemberg and Quadrilatero 2012). While the gastrocnemius and plantaris muscles are recruited for voluntary wheel running, the soleus has higher oxidative capacity and is used both as a postural muscle, and for voluntary wheel running (Pellegrino et al. 2005). Therefore, the fiber‐type composition of these skeletal muscles and muscle recruitment may be important to the response of each muscle to HFD and physical activity.

In addition to the differential effects of exposure to HFD and physical activity on early signaling pathways in soleus, gastrocnemius, and plantaris muscles, this study also found tissue‐specific effects in the liver. It was expected that HFD would lead to increased SOCS3 expression in the liver, and that this increase can be prevented by physical activity (Sarvas et al. 2013). KO SED mice had increased SOCS3 expression compared to all other groups, but this increased SOCS3 was not associated with insulin resistance, as no differences in insulin‐stimulated Akt phosphorylation was observed between groups. However, this finding may highlight impaired lipid metabolism in the liver of KO mice. Both WT RUN and WT SED mice had increased AMPK phosphorylation compared to both KO RUN and KO SED mice. The activation of AMPK in the liver leads to the stimulation of fatty acid oxidation, and the inhibition of lipogenesis (Viollet et al. 2006). The lack of IL‐6 may impair AMPK phosphorylation causing lipid accumulation and increased SOCS3 expression in KO SED mice. While physical activity increases SOCS3 expression in skeletal muscle (Spangenburg et al. 2006), increased SOCS3 expression is induced in the liver, and is linked to the pathogenesis of type 2 diabetes (Sarvas et al. 2013). It was shown that the lack of IL‐6 induced early increases in SOCS3 expression in the liver in response to HFD in sedentary animals. However, in skeletal muscle, physical activity‐induced SOCS3 was exacerbated by the lack of IL‐6, highlighting the tissue‐ and context‐specific differences in SOCS3 expression.

In conclusion, this study utilized the IL‐6 KO model to investigate the role of IL‐6 on HFD‐induced glucose intolerance, and the response to voluntary physical activity in the development of insulin resistance. The collective findings suggest that endogenous IL‐6 is important for the prevention of insulin resistance induced by HFD. This study provides new insight into the role of the IL‐6 in metabolism and energy storage, and highlights tissue‐specific changes in early signaling pathways in response to HFD for 4 weeks. There are many grave pathophysiological outcomes of type 2 diabetes, including increased morbidity and mortality. Importantly, physical activity is effective in the prevention of type 2 diabetes, and is more effective than current pharmacological treatments for humans (Sharoff et al. 2010; Malin et al. 2012). Therefore, it is critical to elucidate the mechanisms behind the increase in insulin sensitivity in response to regular physical activity.

## Conflict of interest

No conflicts of interest, financial or otherwise, are declared by the authors.
